# Prognostic Significance of Altered ATRX/DAXX Gene in Pancreatic Neuroendocrine Tumors: A Meta-Analysis

**DOI:** 10.3389/fendo.2021.691557

**Published:** 2021-06-18

**Authors:** Fei Wang, Xiaowu Xu, Zeng Ye, Yi Qin, Xianjun Yu, Shunrong Ji

**Affiliations:** ^1^ Department of Pancreatic Surgery, Fudan University Shanghai Cancer Center, Shanghai, China; ^2^ Department of Oncology, Shanghai Medical College, Fudan University, Shanghai, China; ^3^ Shanghai Pancreatic Cancer Institute, Shanghai, China; ^4^ Pancreatic Cancer Institute, Fudan University, Shanghai, China

**Keywords:** pancreatic neuroendocrine tumors, ATRX, DAXX, prognosis, meta-analysis

## Abstract

**Background:**

Pancreatic neuroendocrine tumors (PanNETs) are a heterogeneous group of neoplasms with increasing incidence and unpredictable behavior. Whole-exome sequencing recently has shown very frequent somatic mutations in the alpha-thalassemia/mental retardation X-linked (ATRX) and death domain-associated protein (DAXX) genes in PanNETs. And the prognostic significance of altered ATRX/DAXX genes in PanNETs patients have been revealed in several reports. However, many of these include small sample size and hold controversial opinions. To increase statistical power, we performed a systematic review and meta-analysis to determine a pooled conclusion. We examined the impact of altered ATRX/DAXX genes mainly on overall survival (OS), disease-free survival (DFS) and relapse-free survival (RFS) in PanNETs.

**Methods:**

Eligible studies were identified and quality was assessed using multiple search strategies (last search May 2021). Data were collected from studies about prognostic significance of altered ATRX/DAXX in PanNETs. Studies were pooled, and combined hazard ratios (HRs) with 95% confidence intervals (CIs) were used to estimate strength of the associations.

**Results:**

Fourteen studies involving 2313 patients treated for PanNETs were included. After evaluating for publication bias, disease-free survival and relapse-free survival was significantly shortened in patients with altered ATRX/DAXX gene, with combined HR 5.05 (95% confidence interval (CI): 1.58-16.20, *P* = 0.01) and 3.21 (95% confidence interval (CI): 1.44-7.16, *P* < 0.01) respectively. However, the combined data showed there were no difference between patients with altered ATRX/DAXX gene or not in overall survival, with a combined HR 0.71 (95% confidence interval (CI): 0.44-1.15, *P* = 0.23). We also performed a subgroup analysis with metastatic patients in overall survival, showing a combined HR 0.22 (95% confidence interval (CI): 0.11-0.48, *P* = 0.96). The small number of studies and paucity of multivariate analyses are the limitations of our study.

**Conclusions:**

This is the first rigorous pooled analysis assessing ATRX/DAXX mutation as prognostic biomarkers in PanNETs. Patients with altered ATRX/DAXX gene would have poor DFS according to the combined data. And altered ATRX/DAXX genes in metastatic patients showed a trend towards improved overall survival, although the difference did not reach statistical significance.

## Introduction

Pancreatic Neuroendocrine Tumors (PanNETs) are a rare but clinically important form of pancreatic neoplasia (1). Comprising about 3% of all pancreatic tumors, it harbors a significant malignant potential; more than 50% of patients will die of their tumor within 10 years (2). Moreover, the incidence of PanNETs has been statistically significantly increasing, whereas overall survival has remained relatively unchanged over the past several decades (3–5). PanNETs can be broadly classified into functional (hormone producing) and nonfunctional tumors, with a wide spectrum of clinical behaviors. The mainstay of treatment for PanNETs is surgery. However, most patients are contraindicated for resection, because 65% of patients have unresectable or metastatic disease at the time of diagnosis (6). Newer chemotherapeutic agents and molecular targeted therapy have recently been developed to treat the unresectable PanNETs, but their efficacy has been modest (7). Today there is no means by which to select the best therapy for an individual patient. Widely accepted prognostic parameters and systems include tumor size, functional status and World Health Organization (WHO) grade (8, 9). These tools are useful at a population level and currently implemented into routine clinical practice, however it remains difficult to predict at the individual patient level which tumors will recur and behave aggressively (10). Hence, additional markers are needed to improve the prognostic classification of PanNETs.

Recent advances in sequencing technologies have uncovered the molecular basis of numerous cancers that has led to new prognostic classification systems and actionable targets (11). Whole-exome sequencing recently has identified recurrent somatic mutations in the genes DAXX and ATRX in PanNETs (11). The ATRX (alpha-thalassemia/mental retardation syndrome X-linked) and DAXX (death domain associated protein) genes encode proteins bind with each other to form a histone chaperone complex involved in depositing histone variant H3.3 at the telomeres and pericentric heterochromatin regions of the genome (10, 12, 13). Loss of function of either of these proteins results in telomere dysfunction and leads to impaired non-homologous end joining, alternate lengthening of telomeres (ALT) and general genomic instability (14–16). As most frequently mutated genes in sporadic PNETs, the prognostic value of altered ATRX/DAXX is controversial. The result that mutations of ATRX/DAXX were associated with a longer survival was reported by Jiao et al. (1). The same opinion was hold by Raj et al. (17), Park et al. (18) as well as Kim et al. (19). However, others were in disagreement (10, 11, 20–22). In their report, the mutations found in the ATRX/DAXX genes was significantly correlated with a shortened survival time.

For all these reasons, we conducted this meta-analysis, including the large number of studies and patients assessing altered ATRX/DAXX genes in PanNETs to date, and tested both the overall survival (OS), disease-free survival (DFS) and relapse-free survival (RFS) as outcomes. We also performed a subgroup analysis with metastatic patients in overall survival. According to the combined data, patients with altered ATRX/DAXX gene would have prolonged poor DFS and RFS. And altered ATRX/DAXX genes in metastatic patients showed a trend towards improved overall survival, although the difference did not reach statistical significance.

## Materials and Methods

Meta-analysis is the statistical combination of results from two or more separate studies [80]. Therefore, after systematic review and thorough assessment of ATRX/DAXX gene mutations studies in PanNETs, we attempted to perform a pooled analysis of ATRX/DAXX mutations associated with survival in PanNETs in at least three studies.

### Literature Search Strategy

**Figure 1:**
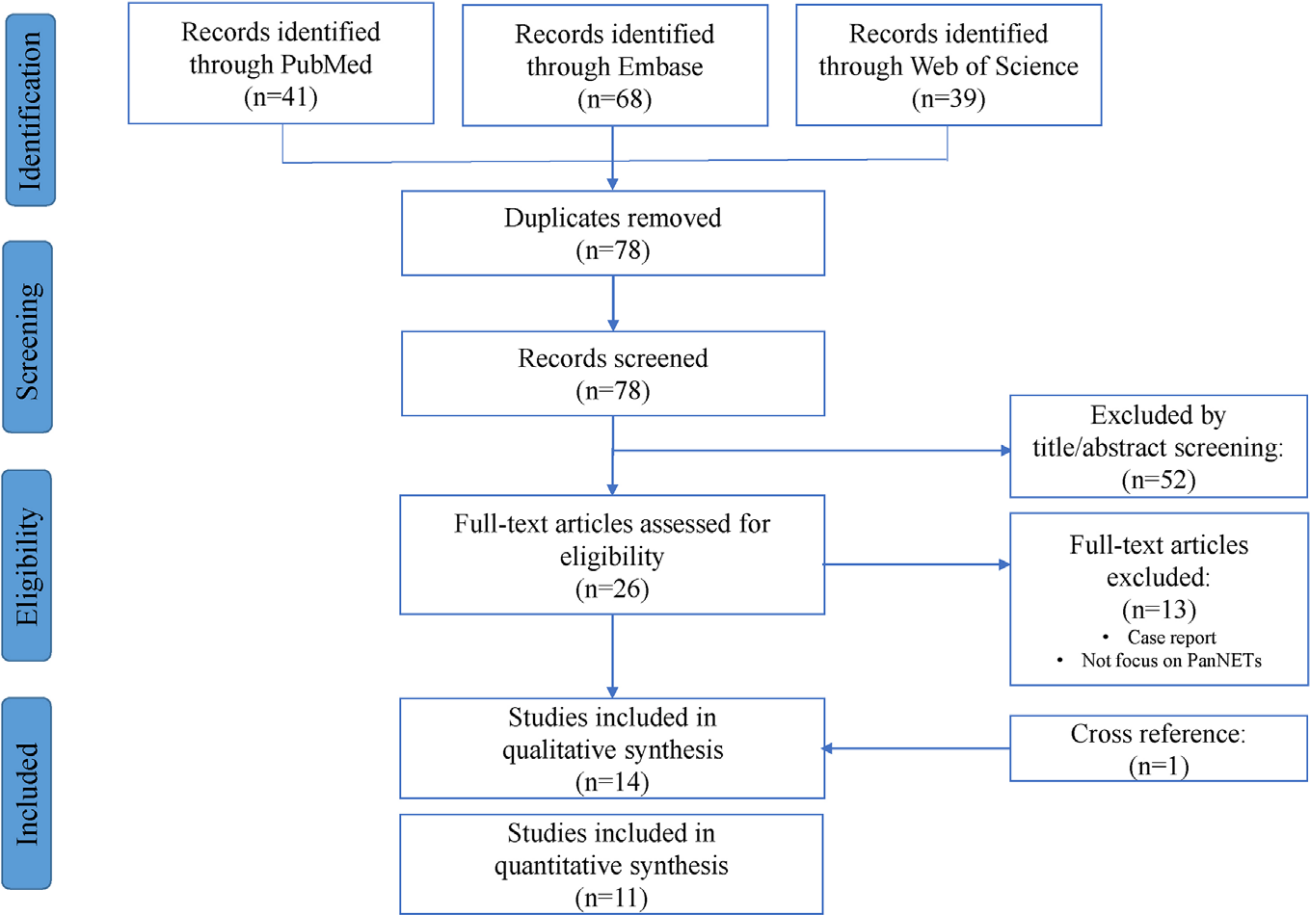
Flow chart of included articles according to the Preferred Reporting Items for Systematic Reviews and Meta-Analyses (PRISMA) statement.

The aim of our search was to identify all primary research articles that assessed the utility of altered ATRX/DAXX as a prognostic factor among individuals treated for PanNETs. A systematic search *via* PubMed, Embase and Web of Science was previously performed on December 2020 and updated in February 2021 and May 2021, in line with the guideline of Preferred Reporting Items for Systematic Reviews and Meta-Analyses (PRISMA) for systematic reviews and meta-analyses by two independent researchers (23). The search strategy was shown in **Appendices A–C** and the identification and selection process for this meta-analysis are illustrated in **Figure 1**. Searches were limited to human articles published in the English language. After removing duplication, a total of 78 publications were identified in the initial literature search. Based on screening of titles or abstracts, 52 records were excluded. Twenty-six articles were left for the further full text assessment. Additionally, reference lists of full-text articles were searched manually for relevant literature and one additional article was found in reference. The search results were analyzed by two independent investigators and any disagreement was resolved by discussion. According to the inclusion and exclusion criteria mentioned below, 13 articles were excluded, and 14 articles were finally included for this meta-analysis. The last search date was 9th May 2021.

### Inclusion and Exclusion Criteria

Prognostic altered ATRX/DAXX studies were considered eligible if they met all of the following initial inclusion criteria: (a) focused on patients under treatment for PanNETs; (b) measured the ATRX/DAXX mutation in tumor; (c) correlated survival outcomes; (d) peer-reviewed journal and (e) published in English **(** **Table 1** **)**. Studies were excluded if they were: (a) case reports, laboratory studies or letters; (b) unpublished data from meeting abstracts; or (c) lacked essential information for the calculation of survival outcomes. Further details about data extraction, statistical analysis, and quality and publication bias assessments can be seen below.

**Table 1:** Criteria for the inclusion articles.

First author	Year	Country	Sample size	Quality assessment	PubMed ID
Yuchen Jiao	2011	USA	68	7	(1)
Angela Chou	2018	Australia	105	8	(10)
Joo Kyung Park	2017	Korea	76	7	(18)
Mauro Cives	2019	Italy	56	7	(21)
Jun Uemura	2019	Japan	100	7	(24)
Nitya Raj	2018	USA	80	7	(17)
Shoki Sato	2014	Japan	16	5	(25)
Ilaria Marinoni	2014	Switzerland	243	7	(26)
Aatur D. Singhi	2019	USA	321	7	(11)
Joo Young Kim	2017	Korea	269	7	(19)
Fei Yuan	2014	China	37	7	(20)
Somak Roy	2018	USA	347	6	(22)
C P Pipinikas	2015	UK	34	6	(27)
Wenzel M Hackeng	2021	Netherlands	561	7	(28)

### Data Extraction

Eligible articles were reviewed independently by two investigators. Disagreements were resolved by consensus and consultation with a third investigator. Data were extracted independently by two authors using a standard protocol. The following information was extracted from each study: PubMed ID; first author; year of publication; number of patients; mutation gene; survival event and hazard ratio (HR) for survival event, as well as their 95% confidence interval (CI) and P value. Multivariate Cox hazard regression analyses were included in our meta-analysis; if these data were not available, univariate hazard regression analyses, log-rank P values or Kaplan–Meier survival curves of survival outcomes were included instead (29). If necessary, HRs were calculated using the available numerical or graphical data and the methods developed by Parmar et al. (30) and Tierney et al. (29). Rounding was adopted when needed. Several corresponding authors of studies were contacted for additional information and data needed for the meta-analytic calculations.

### Quality Assessment

There are various tools for the quality assessment and identification of bias in observational studies (31). In this systematic review, quality of non-randomized studies was determined using the Newcastle–Ottawa scale for cohort studies (32). NOS is comprised of three parameters (eight elements, nine stars total) for quality: selection (four elements, one star each), comparability (one element, up to two stars) and outcome (three elements, one star each). The high-quality choices for each element are marked with a star, and then the number of stars is counted to evaluate the quality of each study. This score has a maximum of nine stars and those studies with ≥7 stars were considered to be of higher quality. Only studies with ≥5 stars were included in the systematic review and subsequent pooled analysis.

### Statistical Analysis

Meta-analyses were carried out using the R studio software (Version 1.3.1056). HRs represent the hazard of death or disease recurrence during the study interval in a patient who had altered ATRX/DAXX genes compared to a patient with wild type. Hazard ratios were considered significant at the *P* < 0.05 level if the 95% CI did not include the value 1. For easy interpretation, HRs were converted to display the contributory effect (e.g., if the survival outcome that ATRX/DAXX mutations bring is more detrimental, then the HR is seen as >1.) Heterogeneity was defined at the I^2^ > 50%. The random-effects model was used if heterogeneity was observed, whilst the fixed-effects model was used in the absence of inter-study heterogeneity. Where possible, heterogeneity was also investigated by sensitivity analysis, which was undertaken by assessing the impact of each study on the combined effect by removing each study sequentially.

### Publication Bias Assessment

Publication bias introduced by researchers only reporting significant positive findings was a concern. Funnel plots with Egger’s bias indicator test were used to assess any reporting or publication bias (33). Funnel plots (i.e., scatterplots demonstrating each study’s effect in relation to their sample size) that are asymmetrical and skewed indicate that bias exists. Egger’s regression intercept provides an estimate of asymmetry of a funnel plot; positive values indicate a trend towards higher levels of test accuracy in studies with smaller sample sizes (34). It was possible to calculate Egger’s regression intercept when data from at least three studies were pooled. When publication bias was identified, we would perform a ‘trim and fill’ re-estimation and sensitivity analysis to assess the potential impact of missing studies (35). A two-tailed *P* value <0.05 was considered significant.

## Results

### Excluded Studies

Our search yielded seventy-eight manuscripts for consideration within this systematic review and subsequent meta-analysis (**Figure 1**). Title and abstract assessment identified twenty-six manuscripts appropriate for evaluation of ATRX/DAXX mutations in PanNETs and full text articles were obtained for these. One additional article was found in reference. On careful review of study methodologies, thirteen reports were excluded and fourteen studies were included in qualitative synthesis.

### Included Studies

Inclusion criteria can be seen in **Table 1**. The full texts of thirteen papers and one from reference (published 2011-2021) fulfilling the criteria for meta-analysis were obtained. We mainly focused on the following efficacy outcomes: Overall survival (OS), defined as the time between the beginning of the study until time of death; disease-free survival (DFS), defined as the time after treatment during which no sign of cancer is found; relapse-free survival (RFS) was defined as the time from commencement of neoadjuvant therapy to disease progression (preoperatively) or recurrence (postoperatively) or death from any cause.

### Study Characteristics

A total of 14 studies with 2313 patients (range from 16-561 per study) were assessed in this meta-analysis (1, 10, 11, 17–22, 24–28). Studies included were published from 2011 to 2021. Among them, 5 studies studied the patients from Asian countries, including Japan (2), South Korea (2), and China (1); 9 studies investigated the patients from European countries and North American countries, including America (4), Australia (1), Italy (1), Switzerland (1), England (1) and Netherlands (1). The main characteristics of these included articles were shown in **Table 2**. Moreover, the results of study quality assessment were also listed in **Table 2**.

**Table 2:** Main characteristics of included articles.

Study design	Prospective or retrospective cohort design with a well-defined study population
Tumor type	Pancreatic Neuroendocrine Tumors (PanNETs)
Gene mutation detection measure	Immunohistochemistry/gene sequencing
Analysis	Reporting of the resulting HRs including 95% CIs or data/Kaplan Meier survival curves to allow their calculation
Survival events	OS/DFS/RFS/other events
PanNET stage/grade	Any
Length of follow-up	Any
Source	Peer-reviewed journals
Language	English

OS, overall survival; DFS, disease-free survival; HR, hazard ratio; CI, confidence interval.

### Meta-Analysis

A summary of the hazard ratios (HRs) estimated from the entire pooled quantitative synthesis for the altered ATRX/DAXX genes can be seen in **Table 3**.

**Table 3:** Characteristics of study enrolled in quantitative synthesis.

First Author	Year	Sample size	Patient cohort	Outcome^#^	Survival events	HR	95%CI		*P* value
Jiao et al. (1)	2011	68	Entire cohort	/	OS	0.4436		0.18 - 1.1	0.0815
Jiao et al. (1)	2011	27	Metastatic cohort	good	OS	0.2222		0.06 -0.84	0.0269
Chou et al. (10)	2018	105	Entire cohort	poor	OS	1.74		0.54-5.59	0.045
Park et al. (18)	2017	76	Entire cohort	good	OS	3.809		1.064–13.630	0.04
Park et al. (18)	2017	68	Metastatic excluded cohort	good	OS	0.11		0.01-0.79	<0.001
Park et al. (18)	2017	15	Metastatic cohort	good	OS	0.24		0.02-2.36	<0.001
Raj et al. (17)	2018	80	Entire cohort*	good	OS	0.44		0.03-5.66	<0.01
Kim et al. (19)	2017	269	Entire cohort	/	OS	1.01		0.46–2.21	0.988
Kim et al. (19)	2017	42	Metastatic cohort	good	OS	0.2		0.07–0.59	0.003
Yuan et al. (20)	2014	37	Entire cohort	poor	OS	4.71		0.00-497.14	<0.05
Park et al. (18)	2017	68	Entire cohort	/	DFS	0.14		0.01-2.05	0.77
Cives et al. (21)	2019	56	Entire cohort	poor	DFS	3.99		0.92-37.39	0.001
Singhi et al. (11)	2017	270	Entire cohort	poor	DFS	11.51		4.85-27.31	<0.001
Roy et al. (22)	2018	292	Entire cohort	poor	DFS	10.17		4.98-20.75	<0.001
Hackeng et al. (28)	2021	561	Entire cohort	poor	RFS	5.144		3.647-7.257	<0.001
Kim et al. (19)	2017	245	Entire cohort	poor	RFS	4.01		2.14–7.50	<0.001
Marinoni et al. (26)	2014	243	Entire cohort	poor	RFS	1.585		1.050-2.400	0.028

Outcome^#^ refers to the survival outcome that altered ATRX/DAXX gene brings; Entire cohort* refers to cohort that all patients in it had metastatic disease.

### The Prognostic Value of ATRX/DAXX Mutation

#### The Prognostic Value in Overall Survival

**Figure 2:**
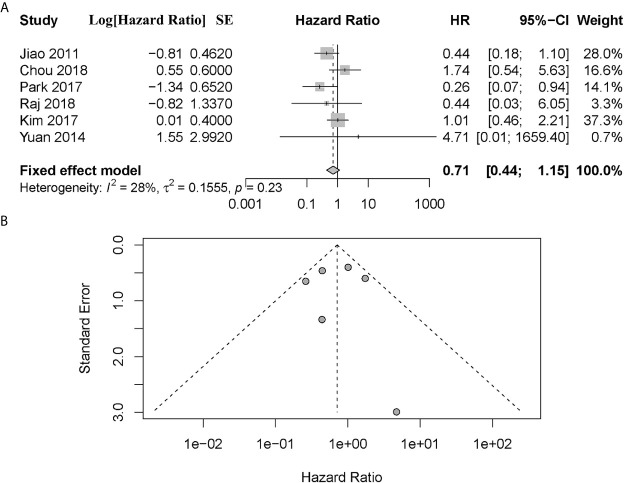
**(A)** Forest plot showing the combined hazard ratio (HR) from studies for the association between ATRX/DAXX mutation and overall survival. Horizontal lines represent 95% confidence interval (CI). Each box represents the HR point estimate, and its area is proportional to the weight of the study, determined by inverse variance weighting. The diamond represents the overall summary estimate, with the 95% CI given by its width. **(B)** Funnel plot of standard error of assessing publication bias.

A total of 6 articles assessed the effect of ATRX/DAXX mutations on the overall survival of PanNETs (1, 10, 17–20). Two of them (1, 19) considered that there was no difference in OS between patients with altered ATRX/DAXX genes or not and the pooled hazard ratio (HR) and 95% confidence interval (CI) supported this view. Two of them (10, 20) showed that ATRX/DAXX mutation was associated with decreased OS, while the rest two studies (17, 18) hold the opposite view. According to the combined data, there was no difference between patients with or without ATRX/DAXX mutation in OS, with a combined HR 0.71 (95% confidence interval (CI): 0.44-1.15, *P* = 0.23) (**Figure 2**). No significant inter-study heterogeneity was found (*P* = 0.23, I^2^ = 28%). There was no evidence of publication bias in these 6 studies (Egger’s test, intercept -0.362, *P* = 0.971).

**Figure 3:**
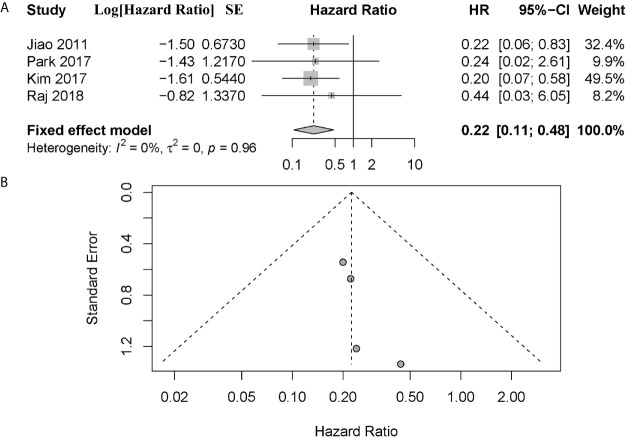
**(A)** Forest plot showing the combined hazard ratio (HR) from subgroup analysis for the association between ATRX/DAXX mutation with metastatic patients and overall survival. Horizontal lines represent 95% confidence interval (CI). Each box represents the HR point estimate, and its area is proportional to the weight of the study, determined by inverse variance weighting. The diamond represents the overall summary estimate, with the 95% CI given by its width. **(B)** Funnel plot of standard error of assessing publication bias.

Notably, four of these six studies performed a subgroup analysis exclusively with metastatic patients (1, 10, 18, 19). And we noted that all patients enrolled in the study of Raj et al. had metastatic disease (17). However, Chou et al. did not provide sufficient information other than *P* value in their study (10). To investigate the prognostic value in metastatic patients and reduce the effect of patient’s disparity, we performed a further meta-analysis exclusively with metastatic patients from these four articles (1, 17–19). The combined data showed that metastatic patients with altered ATRX/DAXX genes would have a better trend in OS but not statistical significance, with a combined HR 0.22 (95% confidence interval (CI): 0.11-0.48, *P* = 0.96) (**Figure 3**). There was no evidence of publication bias in these 4 studies (Egger’s test, intercept -1.968, *P* = 0.155). No significant inter-study heterogeneity was found (*P* = 0.96, I^2^ = 0%).

#### The Prognostic Value in Disease-Free Survival

**Figure 4:**
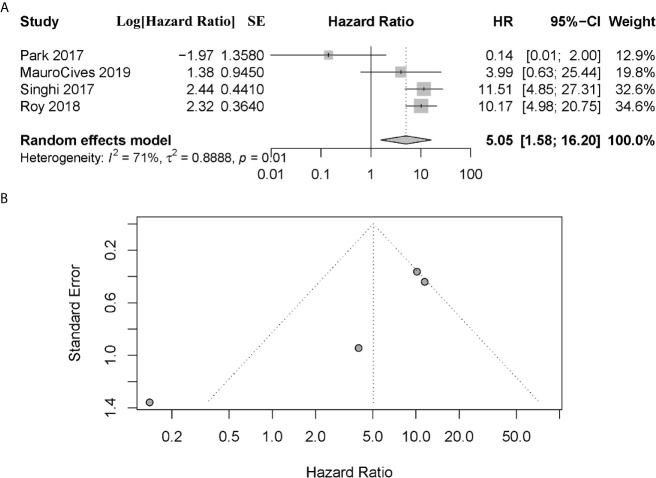
**(A)** Forest plot showing the combined hazard ratio (HR) from studies for the association between ATRX/DAXX mutation and disease-free survival. Horizontal lines represent 95% confidence interval (CI). Each box represents the HR point estimate, and its area is proportional to the weight of the study, determined by inverse variance weighting. The diamond represents the overall summary estimate, with the 95% CI given by its width. **(B)** Funnel plot of standard error of assessing publication bias.

Three of four articles suggested that patients with altered ATRX/DAXX had a worse DFS than the patient with wild type (11, 21, 22). However, Park et al. (18) showed there was no difference in DFS between these patients. The combined data showed that patients with altered ATRX/DAXX would have worse outcome, with a combined HR 5.05 (95% confidence interval (CI): 1.58-16.20, *P* = 0.01) (**Figure 4**). Significant inter-study heterogeneity was found (*P* = 0.01, I^2^ = 71%), so we applied the random-effects model. There was no evidence of publication bias in these studies (Egger’s test, intercept 3.712, *P* = 0.096).

#### The Prognostic Value in Relapse-Free Survival

**Figure 5:**
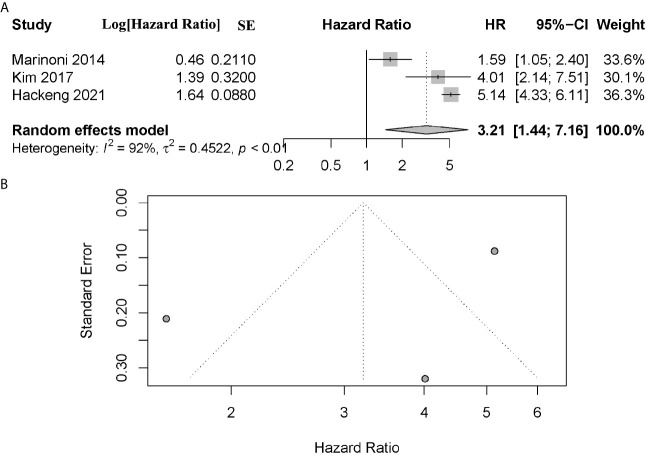
**(A)** Forest plot showing the combined hazard ratio (HR) from studies for the association between ATRX/DAXX mutation and relapse-free survival. Horizontal lines represent 95% confidence interval (CI). Each box represents the HR point estimate, and its area is proportional to the weight of the study, determined by inverse variance weighting. The diamond represents the overall summary estimate, with the 95% CI given by its width. **(B)** Funnel plot of standard error of assessing publication bias.

A total of 3 articles assessed the effect of ATRX/DAXX mutation on the relapse-free survival (RFS) in PanNETs (19, 26, 28). All these authors indicated that patients with ATRX/DAXX mutation had poor outcome in RFS. The combined data showed that patients with altered ATRX/DAXX would have worse outcome, with a combined HR 3.21 (95% confidence interval (CI): 1.44-7.16, *P* < 0.01) (**Figure 5**). Significant inter-study heterogeneity was found (*P* < 0.01, I^2^ = 92%), so we applied the random-effects model. There was no evidence of publication bias in these studies (Egger’s test, intercept 1.950, *P* = 0.5307).

#### The Prognostic Value in Other Survival Events

Cives et al. (21) found altered ATRX/DAXX gene was associated with poor outcome in time to progress (TTP). Marinoni et al. (26) found no difference in tumor-specific survival (TSS) between ATRX/DAXX mutation and ATRX/DAXX normal patients in two independent cohorts. Pipinikas et al. (27) found patients with altered ATRX/DAXX gene had poor outcome in progression free survival (PFS).

### The Prognostic Value in ATRX Mutation

Chou et al. (10) suggested that altered ATRX patients would have poor overall survival. Uemura et al. (24) did not find a significant association between ATRX mutation and OS, but found ATRX mutation patients would have poor RFS. Sato et al. (25) also showed altered ATRX patients would have poor DFS compared to the patients without mutation.

### The Prognostic Value in DAXX Mutation

Both Chou et al. (10) and Uemura et al. (24) showed no difference in OS between patients with altered DAXX or not. In the study by Cives et al. (21), DAXX mutation would lead to worse OS, DFS, TTP and Cancer-specific survival (CSS). Sato et al. (25) also showed patients with altered DAXX would have poor DFS compared to the patients with wide type of DAXX.

### Evaluation of Publication Bias

Publication bias is a well-known problem in meta-analysis (23). As we know, positive results tend to be accepted by journals whilst negative results are often rejected or not even submitted. To avoid publication bias, funnel plots and Egger’s test were both performed in our study. As a result, there was no evaluation of publication bias existing in our analysis.

## Discussion

As the advance in imaging and public awareness in PanNETs, as well as aging population, the incidence of PanNETs has been remarkably increasing, while overall survival has remained relatively unchanged over the past several decades (3–5, 36, 37). Patients with PanNETs usually display a better survival than those with pancreatic ductal adenocarcinomas (38–40). However, PanNETs are still malignant, as evidenced by the fact that the 10-year survival rate for PanNET patients is only 40–50% (2, 9, 41). Current prognostic parameters and systems, such as tumor size and World Health Organization (WHO) grade, are susceptible to interpretation errors, sampling issues and, in a subset of PanNETs, do not accurately reflect the clinical behavior of these neoplasms (22, 42). Hence, additional markers are needed to improve the prognostic classification of PanNETs. In this study, we reviewed several studies revealed the prognostic significance of altered ATRX/DAXX genes in PanNETs.

As for prognostic value in altered ATRX/DAXX genes, there have been opposite opinions on it. Nine previous studies which have found PanNETs with altered ATRX/DAXX are associated with worse outcomes (10, 11, 19–22, 26–28). Two of them were about OS (10, 20), three about DFS (11, 21, 22), three about RFS (19, 26, 28) and one about PFS (27). Marinoni et al. found this association in two independent cohorts consisting of 142 and 101 patients, so did Singhi et al. in a larger cohort of 321 patients (11, 26). In contrast to above findings, 4 studies have suggested that ATRX/DAXX mutation is associated with better survival (1, 17–19). Interestingly, we tried to find the reasons resulting in these discrepant findings. Inspired by Chou et al. (10), we first focused on the cohort differences. For example, in the study of Jiao et al, patients with high stage disease (stage III/IV) were over-represented, and indeed all patients with ATRX/DAXX-negative tumors (28 out of 68) presented with metastatic disease (41% liver metastases) (1). Similarly, all patients in the study of Raj et al. had metastatic disease (17). Of note, both Raj et al. and Jiao et al. (metastatic cohort) as well as Kim et al. (metastatic cohort) showed ATRX/DAXX mutation is of positive prognostic value in overall survival, whereas the study cohort with low percentage of metastatic patients, like Chou et al. (13% liver metastases), presented different results. We assumed that whether ATRX/DAXX loss may be associated with better prognosis in metastatic or advanced disease but worse prognosis in localized tumors. To investigate that assumption, we performed a subgroup meta-analysis with metastatic patients from four studies (1, 17–19).

In metastatic patients, we found that there was a trend for PanNETs with altered ATRX/DAXX to be associated with longer survival. Although this finding did not reach statistical significance (*P* = 0.96), it raises the intriguing possibility that ATRX/DAXX mutation may be associated with poor survival in low stage tumors but better survival in high stage or metastatic tumors. If this association is valid by further studies, we can postulate that PanNETs diagnosed at advanced stage may be molecularly different to those diagnosed at earlier stage and that other cancer pathways may play a more vital role in advanced PanNETs. Ultimately further studies would be required to validate this hypothetical association and to investigate potential mechanisms.

Apart from that, we noted the definition of negative ATRX/DAXX expression in IHC varied among studies, which, to our worry, may contribute to the present discrepant prognostic values. Conventional immunohistochemistry (IHC) is a widely used diagnostic technique in tissue pathology (43). From previous study, we knew that whole-exome sequencing has identified recurrent mutations in the genes DAXX and ATRX, which correlate with loss of protein expression (11, 26). Although the standard of positive staining was generally shared (nuclear staining within tumor cells, using stromal cells as a positive internal control), the definition of negative staining of ATRX/DAXX mutation varied **(** **Supplementary Table S1** **)**. For example, Park et al. (18) defined negative staining as ‘the presence of positive cytoplasmic staining with negative nuclear staining in the presence of positive internal control’. Whereas, Chou et al. considered a negative result as negative nuclear staining in the presence of positive internal control, irrespective of cytoplasmic staining which they interpreted as non-specific and ignored (10). The same definition was shared by Marinoni’s study which demonstrated a high correlation between ATRX/DAXX mutation status and loss of nuclear staining (irrespective of cytoplasmic staining) for ATRX/DAXX and other studies (11, 22, 26). According to different definition, one detection result by IHC may be considered differently (ATRX/DAXX negative or positive expression) and then attributed to different type (ATRX/DAXX mutation or not) so that the conclusion they drew may be influenced. The same worry was brought up by Chou et al. previously, and they postulated the unusually high incidence of ATRX/DAXX-negative (79%) in the patient cohort of Park et al. resulted from the different definition. Then, we focus on the incidence of ATRX/DAXX mutations in the studies we included in quantitative synthesis. The result was listed in **Supplementary Table S2**.

Pancreatic neuroendocrine tumors have been recognized as a distinct clinical entity for nearly a century, yet our understanding of them is incomplete (44). Recently, whole-exome and whole-genome sequencing studies have provided a detailed picture of the underlying genomics of PanNETs (1, 45). With frequent somatic mutations in PanNETs, ATRX and DAXX have received renewed attention. ATRX was first discovered in the a-thalassemia, mental retardation, X-linked (ATRX) syndrome, and DAXX was originally identified as a specifical Fas death receptor binding protein enhancing Fas-mediated apoptosis and activating the Jun N-terminal kinase (JNK) pathway (46, 47). Somatic inactivating mutations in ATRX and DAXX are frequent in PanNETs, mutually exclusive, proteins encoded by these two genes bind with each other to form a histone chaperone complex involved in depositing histone variant H3.3 at the telomeres and pericentric heterochromatin regions of the genome (12, 13). Thus, loss of function of either of these two proteins results in telomere dysfunction and leads to impaired non-homologous end joining, alternate lengthening of telomeres (ALT) as well as general genomic instability (14–16). Apart from ATRX/DAXX, somatic mutations of MEN1, mammalian target of rapamycin (mTOR) pathway genes including PTEN, PIK3CA, and TSC2, genes involved in DNA damage repair such as MUTHY, and genes involved in chromatin modification have been reported as commonly mutated genes in pathogenesis and development of PanNETs (1, 45).

As far as we know, for the first time, we assessed the significance of altered ATRX/DAXX genes for PanNETs in a meta-analysis. Based on the combined HR and 95% CI, we believe that ATRX/DAXX mutation is associated with poor DFS and there is no difference between ATRX/DAXX wild type patients and altered patients in OS. However, one should keep in mind the following limitations. Firstly, some articles only provide Kaplan-Meier curve but not HR or follow-up data. Though the method of HR extrapolation from survival curves has been previously validated (30), there may have been errors due to inaccurate readings, increasing the deviation from the original data. Secondly, many other factors might be responsible for the overall survival and other survival events. However, due to the lack of sufficient articles or effective data, these factors were not assessed in our study. Thirdly, our pooled analysis about the prognostic significance of DAXX/ATRX mutation in DFS (I^2^ = 71) and RFS (I^2^ = 92) was with significant inter-study heterogeneity. To conclude, patients with altered ATRX/DAXX gene would have poor DFS and RFS according to the combined data. And there was no difference in OS between patients with ATRX/DAXX mutation or not. Additionally, metastatic patients with altered ATRX/DAXX genes in metastatic patients showed a trend towards improved overall survival, although the difference did not reach statistical significance.

## Data Availability Statement

The original contributions presented in the study are included in the article/**Supplementary Material**. Further inquiries can be directed to the corresponding authors.

## Author Contributions

Study concept and design: SJ, XX, and FW. Acquisition of data: SJ, FW, and ZY. Analysis and interpretation of data: FW, XX, and SJ. Drafting of the manuscript: FW and SJ. Revision of manuscript: XX, XY, SJ, ZY, and YQ. All authors contributed to the article and approved the submitted version.

## Funding

This work was jointly supported by National Natural Science Foundation [81972250, 81871950 and 81972725], the National Science Foundation for Distinguished Young Scholars of China [81625016]. Clinical and Scientific Innovation Project of Shanghai Hospital Development Center (SHDC12018109).

## Conflict of Interest

The authors declare that the research was conducted in the absence of any commercial or financial relationships that could be construed as a potential conflict of interest.

## References

[1] Jiao Y Shi C Edil BH de Wilde RF Klimstra DS Maitra A. DAXX/ATRX, MEN1, and mTOR Pathway Genes Are Frequently Altered in Pancreatic Neuroendocrine Tumors. Science (2011) 331(6021):1199–203. 10.1126/science.1200609 PMC3144496 21252315

[2] Halfdanarson TR Rubin J Farnell MB Grant CS Petersen GM. Pancreatic Endocrine Neoplasms: Epidemiology and Prognosis of Pancreatic Endocrine Tumors. Endocr Relat Cancer (2008) 15(2):409–27. 10.1677/ERC-07-0221 PMC2693313 18508996

[3] Franko J Feng W Yip L Genovese E Moser AJ. Non-Functional Neuroendocrine Carcinoma of the Pancreas: Incidence, Tumor Biology, and Outcomes in 2,158 Patients. J Gastrointest Surg (2010) 14(3):541–8. 10.1007/s11605-009-1115-0 19997980

[4] Halfdanarson TR Rabe KG Rubin J Petersen GM. Pancreatic Neuroendocrine Tumors (PNETs): Incidence, Prognosis and Recent Trend Toward Improved Survival. Ann Oncol (2008) 19(10):1727–33. 10.1093/annonc/mdn351 PMC2735065 18515795

[5] Yao JC Hassan M Phan A Dagohoy C Leary C Mares JE. One Hundred Years After “Carcinoid”: Epidemiology of and Prognostic Factors for Neuroendocrine Tumors in 35,825 Cases in the United States. J Clin Oncol (2008) 26(18):3063–72. 10.1200/JCO.2007.15.4377 18565894

[6] Valle JW Eatock M Clueit B Gabriel Z Ferdinand R Mitchell S. A Systematic Review of Non-Surgical Treatments for Pancreatic Neuroendocrine Tumours. Cancer Treat Rev (2014) 40(3):376–89. 10.1016/j.ctrv.2013.08.007 24296109

[7] Sharma J Duque M Saif MW. Emerging Therapies and Latest Development in the Treatment of Unresectable Pancreatic Neuroendocrine Tumors: An Update for Clinicians. Therap Adv Gastroenterol (2013) 6(6):474–90. 10.1177/1756283X13498808 PMC3808571 24179483

[8] Reid MD Bagci P Ohike N Saka B Erbarut Seven I Dursun N. Calculation of the Ki67 Index in Pancreatic Neuroendocrine Tumors: A Comparative Analysis of Four Counting Methodologies. Mod Pathol (2015) 28(5):686–94. 10.1038/modpathol.2014.156 PMC4460192 25412850

[9] Ekeblad S Skogseid B Dunder K Oberg K Eriksson B. Prognostic Factors and Survival in 324 Patients With Pancreatic Endocrine Tumor Treated at a Single Institution. Clin Cancer Res (2008) 14(23):7798–803. 10.1158/1078-0432.Ccr-08-0734 19047107

[10] Chou A Itchins M de Reuver PR Arena J Clarkson A Sheen A. ATRX Loss Is an Independent Predictor of Poor Survival in Pancreatic Neuroendocrine Tumors. Hum Pathol (2018) 82:249–57. 10.1016/j.humpath.2018.07.032 30081149

[11] Singhi AD Liu TC Roncaioli JL Cao D Zeh HJ Zureikat AH. Alternative Lengthening of Telomeres and Loss of DAXX/ATRX Expression Predicts Metastatic Disease and Poor Survival in Patients With Pancreatic Neuroendocrine Tumors. Clin Cancer Res (2017) 23(2):600–9. 10.1158/1078-0432.CCR-16-1113 PMC6560642 27407094

[12] Goldberg AD Banaszynski LA Noh KM Lewis PW Elsaesser SJ Stadler S. Distinct Factors Control Histone Variant H3.3 Localization at Specific Genomic Regions. Cell (2010) 140(5):678–91. 10.1016/j.cell.2010.01.003 PMC2885838 20211137

[13] Lewis PW Elsaesser SJ Noh KM Stadler SC Allis CD. Daxx is an H3.3-Specific Histone Chaperone and Cooperates With ATRX in Replication-Independent Chromatin Assembly at Telomeres. Proc Natl Acad Sci U S A (2010) 107(32):14075–80. 10.1073/pnas.1008850107 PMC2922592 20651253

[14] Watson LA Goldberg H Bérubé NG. Emerging Roles of ATRX in Cancer. Epigenomics (2015) 7(8):1365–78. 10.2217/epi.15.82 26646632

[15] Lovejoy CA Li W Reisenweber S Thongthip S Bruno J de Lange T. Loss of ATRX, Genome Instability, and an Altered DNA Damage Response are Hallmarks of the Alternative Lengthening of Telomeres Pathway. PloS Genet (2012) 8(7):e1002772. 10.1371/journal.pgen.1002772 22829774 PMC3400581

[16] Clynes D Jelinska C Xella B Ayyub H Scott C Mitson M. Suppression of the Alternative Lengthening of Telomere Pathway by the Chromatin Remodelling Factor ATRX. Nat Commun (2015) 6:7538. 10.1038/ncomms8538 26143912 PMC4501375

[17] Raj N Shah R Stadler Z Mukherjee S Chou J Untch B. Real-Time Genomic Characterization of Metastatic Pancreatic Neuroendocrine Tumors Has Prognostic Implications and Identifies Potential Germline Actionability. Jco Precis Oncol (2018) 2018:1–18. 10.1200/PO.17.00267 PMC6345401 30687805

[18] Park JK Paik WH Lee K Ryu JK Lee SH Kim YT. DAXX/ATRX and MEN1 Genes are Strong Prognostic Markers in Pancreatic Neuroendocrine Tumors. Oncotarget (2017) 8(30):49796–806. 10.18632/oncotarget.17964 PMC5564808 28591701

[19] Kim JY Brosnan-Cashman JA An S Kim SJ Song KB Kim MS. Alternative Lengthening of Telomeres in Primary Pancreatic Neuroendocrine Tumors Is Associated With Aggressive Clinical Behavior and Poor Survival. Clin Cancer Res (2017) 23(6):1598–606. 10.1158/1078-0432.CCR-16-1147 PMC5354973 27663587

[20] Yuan F Shi M Ji J Shi H Zhou C Yu Y. KRAS and DAXX/ATRX Gene Mutations are Correlated With the Clinicopathological Features, Advanced Diseases, and Poor Prognosis in Chinese Patients With Pancreatic Neuroendocrine Tumors. Int J Biol Sci (2014) 10(9):957–65. 10.7150/ijbs.9773 PMC4159686 25210493

[21] Cives M Partelli S Palmirotta R Lovero D Mandriani B Quaresmini D. DAXX Mutations as Potential Genomic Markers of Malignant Evolution in Small Nonfunctioning Pancreatic Neuroendocrine Tumors. Sci Rep (2019) 9(1):18614. 10.1038/s41598-019-55156-0 31819132 PMC6901561

[22] Roy S LaFramboise WA Liu TC Cao D Luvison A Miller C. Loss of Chromatin-Remodeling Proteins and/or CDKN2A Associates With Metastasis of Pancreatic Neuroendocrine Tumors and Reduced Patient Survival Times. Gastroenterology (2018) 154(8):2060–3 e8. 10.1053/j.gastro.2018.02.026 29486199 PMC5985217

[23] Thornton A Lee P. Publication Bias in Meta-Analysis: its Causes and Consequences. J Clin Epidemiol (2000) 53(2):207–16. 10.1016/s0895-4356(99)00161-4 10729693

[24] Uemura J Okano K Oshima M Suto H Ando Y Kumamoto K. Immunohistochemically Detected Expression of ATRX, TSC2, and PTEN Predicts Clinical Outcomes in Patients With Grade 1 and 2 Pancreatic Neuroendocrine Tumors. Ann Surg (2019). 10.1097/SLA.0000000000003624 31599805

[25] Sato S Tsuchikawa T Nakamura T Sato N Tamoto E Okamura K. Impact of the Tumor Microenvironment in Predicting Postoperative Hepatic Recurrence of Pancreatic Neuroendocrine Tumors. Oncol Rep (2014) 32(6):2753–9. 10.3892/or.2014.3530 25310565

[26] Marinoni I Kurrer AS Vassella E Dettmer M Rudolph T Banz V. Loss of DAXX and ATRX Are Associated With Chromosome Instability and Reduced Survival of Patients With Pancreatic Neuroendocrine Tumors. Gastroenterology (2014) 146(2):453–60.e5. 10.1053/j.gastro.2013.10.020 24148618

[27] Pipinikas CP Dibra H Karpathakis A Feber A Novelli M Oukrif D. Epigenetic Dysregulation and Poorer Prognosis in DAXX-deficient Pancreatic Neuroendocrine Tumours. Endocr Relat Cancer (2015) 22(3):L13–8. 10.1530/ERC-15-0108 PMC4496774 25900181

[28] Hackeng WM Brosens LAA Kim JY O’Sullivan R Sung Y-N Liu T-C. Non-Functional Pancreatic Neuroendocrine Tumours: ATRX/DAXX and Alternative Lengthening of Telomeres (ALT) Are Prognostically Independent From ARX/PDX1 Expression and Tumour Size. Gut (2021). 10.1136/gutjnl-2020-322595 PMC8511349 33849943

[29] Tierney JF Stewart LA Ghersi D Burdett S Sydes MR. Practical Methods for Incorporating Summary Time-to-Event Data Into Meta-Analysis. Trials (2007) 8:16. 10.1186/1745-6215-8-16 17555582 PMC1920534

[30] Parmar MK Torri V Stewart L. Extracting Summary Statistics to Perform Meta-Analyses of the Published Literature for Survival Endpoints. Stat Med (1998) 17(24):2815–34. 10.1002/(sici)1097-0258(19981230)17:24<2815::aid-sim110>3.0.co;2-8 9921604

[31] Sanderson S Tatt ID Higgins JP. Tools for Assessing Quality and Susceptibility to Bias in Observational Studies in Epidemiology: A Systematic Review and Annotated Bibliography. Int J Epidemiol (2007) 36(3):666–76. 10.1093/ije/dym018 17470488

[32] Stang A. Critical Evaluation of the Newcastle-Ottawa Scale for the Assessment of the Quality of Nonrandomized Studies in Meta-Analyses. Eur J Epidemiol (2010) 25(9):603–5. 10.1007/s10654-010-9491-z 20652370

[33] Egger M Davey Smith G Schneider M Minder C. Bias in Meta-Analysis Detected by a Simple, Graphical Test. BMJ (1997) 315(7109):629–34. 10.1136/bmj.315.7109.629 PMC2127453 9310563

[34] Song F Khan KS Dinnes J Sutton AJ. Asymmetric Funnel Plots and Publication Bias in Meta-Analyses of Diagnostic Accuracy. Int J Epidemiol (2002) 31(1):88–95. 10.1093/ije/31.1.88 11914301

[35] Duval S Tweedie R. Trim and Fill: A Simple Funnel-Plot-Based Method of Testing and Adjusting for Publication Bias in Meta-Analysis. Biometrics (2000) 56(2):455–63. 10.1111/j.0006-341x.2000.00455.x 10877304

[36] Strosberg JR Cheema A Weber JM Ghayouri M Han G Hodul PJ. Relapse-Free Survival in Patients With Nonmetastatic, Surgically Resected Pancreatic Neuroendocrine Tumors: An Analysis of the AJCC and ENETS Staging Classifications. Ann Surg (2012) 256(2):321–5. 10.1097/SLA.0b013e31824e6108 22415420

[37] Zhang J Francois R Iyer R Seshadri M Zajac-Kaye M Hochwald SN. Current Understanding of the Molecular Biology of Pancreatic Neuroendocrine Tumors. J Natl Cancer Inst (2013) 105(14):1005–17. 10.1093/jnci/djt135 PMC6281020 23840053

[38] Fesinmeyer MD Austin MA Li CI De Roos AJ Bowen DJ. Differences in Survival by Histologic Type of Pancreatic Cancer. Cancer Epidemiol Biomarkers Prev (2005) 14(7):1766–73. 10.1158/1055-9965.Epi-05-0120 16030115

[39] Bilimoria KY Talamonti MS Tomlinson JS Stewart AK Winchester DP Ko CY. Prognostic Score Predicting Survival After Resection of Pancreatic Neuroendocrine Tumors: Analysis of 3851 Patients. Ann Surg (2008) 247(3):490–500. 10.1097/SLA.0b013e31815b9cae 18376195

[40] Jarufe NP Coldham C Orug T Mayer AD Mirza DF Buckels JA. Neuroendocrine Tumours of the Pancreas: Predictors of Survival After Surgical Treatment. Dig Surg (2005) 22(3):157–62. 10.1159/000087148 16043962

[41] Hochwald SN Zee S Conlon KC Colleoni R Louie O Brennan MF. Prognostic Factors in Pancreatic Endocrine Neoplasms: An Analysis of 136 Cases With a Proposal for Low-Grade and Intermediate-Grade Groups. J Clin Oncol (2002) 20(11):2633–42. 10.1200/jco.2002.10.030 12039924

[42] Kuo EJ Salem RR. Population-Level Analysis of Pancreatic Neuroendocrine Tumors 2 cm or Less in Size. Ann Surg Oncol (2013) 20(9):2815–21. 10.1245/s10434-013-3005-7 23771245

[43] Tan WCC Nerurkar SN Cai HY Ng HHM Wu D Wee YTF. Overview of Multiplex Immunohistochemistry/Immunofluorescence Techniques in the Era of Cancer Immunotherapy. Cancer Commun (Lond) (2020) 40(4):135–53. 10.1002/cac2.12023 PMC7170662 32301585

[44] Scott AT Weitz M Breheny PJ Ear PH Darbro B Brown BJ. Gene Expression Signatures Identify Novel Therapeutics for Metastatic Pancreatic Neuroendocrine Tumors. Clin Cancer Res (2020) 26(8):2011–21. 10.1158/1078-0432.CCR-19-2884 PMC7165057 31937620

[45] Scarpa A Chang DK Nones K Corbo V Patch AM Bailey P. Whole-Genome Landscape of Pancreatic Neuroendocrine Tumours. Nature (2017) 543(7643):65–71. 10.1038/nature21063 28199314

[46] Gibbons RJ Pellagatti A Garrick D Wood WG Malik N Ayyub H. Identification of Acquired Somatic Mutations in the Gene Encoding Chromatin-Remodeling Factor ATRX in the Alpha-Thalassemia Myelodysplasia Syndrome (ATMDS). Nat Genet (2003) 34(4):446–9. 10.1038/ng1213 12858175

[47] Yang X Khosravi-Far R Chang HY Baltimore D. Daxx, a Novel Fas-binding Protein That Activates JNK and Apoptosis. Cell (1997) 89(7):1067–76. 10.1016/s0092-8674(00)80294-9 PMC2989411 9215629

