# Supplementary material for: Prognostic Significance of Altered ATRX/DAXX Gene in Pancreatic Neuroendocrine Tumors: A Meta-Analysis
Source: Front Endocrinol (Lausanne). 2021 Jun 18;12:691557. doi: 10.3389/fendo.2021.691557 (PMC8253224; doi:10.3389/fendo.2021.691557)
Supplement: Supplementary file 1 [file Table_1.docx]

**Supplementary Table S1** Immunohistochemistry criteria of negative staining

| First author | Sample type | Negative scoring criterion |
| --- | --- | --- |
| Jiao (1) | FFPE | N |
| Marinoni (23) | FFPE | A |
| Sato (25) | FFPE | B |
| Pipinikas (24) | FFPE | N |
| Singhi (11) | FFPE | A |
| Kim (19) | FFPE | B |
| Park (18) | FFPE | B |
| Roy (22) | FFPE | A |
| Chou (10) | FFPE | A |
| Uemura (26) | FFPE | A |
| Hackeng (27) | FFPE | B |

A refers to negative nuclear staining in the presence of positive internal control, irrespective of cytoplasmic staining; B refers to the presence of positive cytoplasmic staining with negative nuclear staining in the presence of positive internal control; N refers to the negative scoring criterion not clearly stated in that study.

**Supplementary Table S2** The incidence of ATRX/DAXX mutations

| PubMed ID | First author | Sample size | Detection measure | Mutation in ATRX/DAXX | |
| --- | --- | --- | --- | --- | --- |
| (1) | Jiao | 68 | IHC | | 43% |
| (10) | Chou | 105 | IHC | | 25% |
| (18) | Park | 76 | IHC | | 79% |
| (17) | Raj | 80 | GS | | 65% |
| (19) | Kim | 269 | IHC | | 19% |
| (20) | Yuan | 37 | GS | | 54% |
| (21) | Cives | 56 | GS | | 20% |
| (11) | Singhi | 270 | IHC | | 26% |
| (22) | Roy | 292 | IHC | | 23% |
| (27) | Hackeng | 561 | IHC | | 25% |
| (23) | Marinoni | 243 | IHC | | 25% |

Abbreviations: IHC, Immunohistochemistry; GS, Gene sequencing.
