# Supplementary material for: Prognostic Significance of Altered ATRX/DAXX Gene in Pancreatic Neuroendocrine Tumors: A Meta-Analysis
Source: Front Endocrinol (Lausanne). 2021 Jun 18;12:691557. doi: 10.3389/fendo.2021.691557 (PMC8253224; doi:10.3389/fendo.2021.691557)
Supplement: Supplementary file 2 [file DataSheet_1.docx]

**Appendix A**

PubMed search query: ((((ATRX[Title/Abstract]) OR ("ATRX protein, human" [Supplementary Concept])) OR ((DAXX[Title/Abstract]) OR ("DAXX protein, human" [Supplementary Concept]))) AND (((((((((((((pancreatic neuroendocrine tumor[Title/Abstract]) OR (Neuroendocrine pancreatic tumor[Title/Abstract])) OR (Pancreatic neuroendocrine tumors[Title/Abstract])) OR (pancreatic neuroendocrine carcinogenesis[Title/Abstract])) OR (pancreatic neuroendocrine neoplasms[Title/Abstract])) OR (("Adenoma, Islet Cell"[Mesh]) OR ((((((((((((((((Adenomas, Islet Cell[Title/Abstract]) OR (Islet Cell Adenoma[Title/Abstract])) OR (Islet Cell Adenomas[Title/Abstract])) OR (Islet Cell Tumor[Title/Abstract])) OR (Islet Cell Tumors[Title/Abstract])) OR (Tumor, Islet Cell[Title/Abstract])) OR (Tumors, Islet Cell[Title/Abstract])) OR (Langerhans Tumor Islet[Title/Abstract])) OR (Tumor Islet, Langerhans[Title/Abstract])) OR (Island Cell Tumor[Title/Abstract])) OR (Island Cell Tumors[Title/Abstract])) OR (Tumor, Island Cell[Title/Abstract])) OR (Tumors, Island Cell[Title/Abstract])) OR (Pancreatic Islet Cell Tumors[Title/Abstract])) OR (Nesidioblastoma[Title/Abstract])) OR (Nesidioblastomas[Title/Abstract])))) OR (("Insulinoma"[Mesh]) OR (((((((((((((Insulinomas[Title/Abstract]) OR (Adenoma, beta-Cell[Title/Abstract])) OR (Adenoma, beta Cell[Title/Abstract])) OR (Adenomas, beta-Cell[Title/Abstract])) OR (beta-Cell Adenoma[Title/Abstract])) OR (beta-Cell Adenomas[Title/Abstract])) OR (Insuloma[Title/Abstract])) OR (Insulomas[Title/Abstract])) OR (beta-Cell Tumor[Title/Abstract])) OR (Tumor, beta-Cell[Title/Abstract])) OR (Tumors, beta-Cell[Title/Abstract])) OR (beta Cell Tumor[Title/Abstract])) OR (beta-Cell Tumors[Title/Abstract])))) OR (("Gastrinoma"[Mesh]) OR ((((((((Gastrinomas[Title/Abstract]) OR (Islet Cell Tumor, Ulcerogenic[Title/Abstract])) OR (Ulcerogenic Islet Cell Tumor[Title/Abstract])) OR (Gastrin-Producing Tumor[Title/Abstract])) OR (Gastrin Producing Tumor[Title/Abstract])) OR (Gastrin-Producing Tumors[Title/Abstract])) OR (Tumor, Gastrin-Producing[Title/Abstract])) OR (Tumors, Gastrin-Producing[Title/Abstract])))) OR (("Somatostatinoma"[Mesh]) OR (Somatostatinomas[Title/Abstract]))) OR (("Glucagonoma"[Mesh]) OR (((((((((((((((Glucagonomas[Title/Abstract]) OR (alpha-Cell Tumor[Title/Abstract])) OR (Tumor, alpha-Cell[Title/Abstract])) OR (Tumors, alpha-Cell[Title/Abstract])) OR (alpha Cell Tumor[Title/Abstract])) OR (alpha-Cell Tumors[Title/Abstract])) OR (Glucagonoma Syndrome[Title/Abstract])) OR (Glucagonoma Syndromes[Title/Abstract])) OR (Syndrome, Glucagonoma[Title/Abstract])) OR (Syndromes, Glucagonoma[Title/Abstract])) OR (Adenoma, alpha-Cell[Title/Abstract])) OR (Adenoma, alpha Cell[Title/Abstract])) OR (Adenomas, alpha-Cell[Title/Abstract])) OR (alpha-Cell Adenoma[Title/Abstract])) OR (alpha-Cell Adenomas[Title/Abstract])))) OR (("Vipoma"[Mesh]) OR ((((((((((((((((((((((((((Vipomas[Title/Abstract]) OR (Diarrheogenic Tumor[Title/Abstract])) OR (Diarrheogenic Tumors[Title/Abstract])) OR (Tumor, Diarrheogenic[Title/Abstract])) OR (Tumors, Diarrheogenic[Title/Abstract])) OR (Pancreatic VIPoma[Title/Abstract])) OR (Pancreatic VIPomas[Title/Abstract])) OR (VIPoma, Pancreatic[Title/Abstract])) OR (VIPomas, Pancreatic[Title/Abstract])) OR (Vasoactive Intestinal Peptide-Producing Tumor[Title/Abstract])) OR (Vasoactive Intestinal Peptide Producing Tumor[Title/Abstract])) OR (Diarrheogenic Islet Cell Tumor[Title/Abstract])) OR (Vasoactive Intestinal Peptide (VIP) Tumor[Title/Abstract])) OR (Pancreatic Cholera[Title/Abstract])) OR (Cholera, Pancreatic[Title/Abstract])) OR (Vipoma Syndrome[Title/Abstract])) OR (Syndrome, Vipoma[Title/Abstract])) OR (Watery Diarrhea Syndrome[Title/Abstract])) OR (Watery Diarrhea, Hypokalemia,[Title/Abstract] OR Achlorhydria Syndrome[Title/Abstract])) OR (WDHA[Title/Abstract])) OR (WDHH[Title/Abstract])) OR (WDHA Syndrome[Title/Abstract])) OR (WDHA Syndromes[Title/Abstract])) OR (Verner-Morrison Syndrome[Title/Abstract])) OR (Syndrome, Verner-Morrison[Title/Abstract])) OR (Verner Morrison Syndrome[Title/Abstract])))) OR ((ectopic adrenocorticotropic hormone secreting adenoma[Title/Abstract]) OR (ACTHoma[Title/Abstract]))))) AND (((("Prognosis"[Mesh]) OR ((((Prognostic Factors[Title/Abstract]) OR (Factor, Prognostic[Title/Abstract])) OR (Factors, Prognostic[Title/Abstract])) OR (Prognostic Factor[Title/Abstract]))) OR (prognos*[Title/Abstract])))

**Appendix B**

Embase search query: (daxx:ab,ti OR atrx:ab,ti OR 'atrx gene':ab,ti OR 'atrx protein human':ab,ti OR 'daxx gene':ab,ti OR 'daxx protein human':ab,ti) AND ('pancreatic neuroendocrine tumor':ab,ti OR 'neuroendocrine pancreatic tumor':ab,ti OR 'pancreatic neuroendocrine tumors':ab,ti OR 'pancreatic neuroendocrine carcinogenesis':ab,ti OR 'pancreatic neuroendocrine neoplasms':ab,ti) AND (prognosis:ab,ti OR 'prognostic value':ab,ti OR prognos*:ab,ti)

**Appendix C**

Web of Science search query: AB= (DAXX OR ATRX) AND AB= (pancreatic neuroendocrine tumor OR Neuroendocrine pancreatic tumor OR Pancreatic neuroendocrine tumors OR pancreatic neuroendocrine carcinogenesis OR pancreatic neuroendocrine neoplasms) AND AB= (Prognosis OR Prognostic Factors OR prognos* OR Prognostic Factor)
